# Integration of CSF pressure and flow data to further investigate nature of cerebrospinal compliance in hydrocephalus

**DOI:** 10.1186/2045-8118-12-S1-O66

**Published:** 2015-09-18

**Authors:** Simon Garnotel, Eric Schmidt, Zofi Czosnyka, Marc Baroncini, Gwenaël Pagé, Olivier Balédent

**Affiliations:** 1 University of Picardie Jules Verne, BioFlow Image Laboratory, Amiens, France; 2 University hospital of Toulouse, Neurosciences Department, Toulouse, France; 3 University of Cambridge, Department of Clinical Neurosciences, Cambridge, UK; 4 University hostiptal of Lille, INSERM U837, Jean-Pierre AUbert Research Center, Lille, France

## Introduction

Intracranial pressure (ICP) monitoring and infusion test are widely used in the diagnosis and management of hydrocephalus. MR neuroimaging allows the quantification of cerebrospinal fluid (CSF) and blood flow dynamics that can be used to calculate intracranial volume changes (IVC) during cardiac cycle and to help diagnose active hydrocephalus which need the placement of a shunt. Since compliance is a function of ICP and IVC changes, the aim of this work is to combine these two techniques to calculate the cerebrospinal system compliance along the cardiac cycle.

## Methods

36 patients with suspected hydrocephalus underwent a spinal infusion test: a constant rate infusion of 1 mL/min was administered until pressure reached a specific threshold. Pressure sensor data was recorded with ICM+. Previously all patients had a morphological MRI, in which two PC-MRI measurements were performed to quantify the dynamics of CSF and cerebral arterial and venous flows, which move through the cranium during the cardiac cycle. These flows have been extracted by the homemade software Flow Analysis.

A homemade “ICP/flow analysis” software was developed to calculate the mean ICP cycle over the cardiac cycle in four periods of the infusion procedure (basal, up, plateau, down) from ICM+ extracted data. It calculates the IVC curve during the cardiac cycle, which generates ICP changes, from Flow Analysis extracted data and determines the compliance change of the cerebrospinal system during cardiac cycle, function of the previous mean ICP and IVC results.

## Results

The ICP/flow analysis software was used on the 36 patients, in whom the final clinical diagnosis was still unknown. For the different periods of the infusion test, the amplitudes of ICP were: 12±3 mmHg for basal, 24±4 for up, 32±6 for plateau, 24±5 for decrease of ICP after infusion. From flow MRI, IVC was equal to 683±341 mm^3^. The mean values of the compliance in the cardiac cycle over all the patients were equal to 476±292 and 120±110 mm^3^/mmHg during the basal and the plateau period, respectively. These results show significant compliance variability over the cardiac cycle, especially a decrease in its value in the middle of the cycle, when volume and pressure are increased. An exponential correlation was found between the mean value of compliance and ICP amplitude during basal and plateau periods with a correlation coefficient of 0.85.

## Conclusions

A multi-platform ICP/flow analysis software was performed to combine ICP, CSF and cerebral blood flow changes during a short time of a cardiac cycle. These results create a new point of view on the cerebrospinal system compliance, showing that it is not constant during the cardiac cycle and that significant variability exists in patients suffering from NPH.

